# The top 100 most cited papers in insomnia: A bibliometric analysis

**DOI:** 10.3389/fpsyt.2022.1040807

**Published:** 2023-01-04

**Authors:** Qingyun Wan, Kai Liu, Xiaoqiu Wang, Shuting Luo, Xiya Yuan, Chi Wang, Jing Jiang, Wenzhong Wu

**Affiliations:** Department of Acupuncture and Rehabilitation, Jiangsu Province Hospital of Chinese Medicine, Affiliated Hospital of Nanjing University of Chinese Medicine, Nanjing, Jiangsu, China

**Keywords:** bibliometric analysis, citations, insomnia, trends, VOSviewer

## Abstract

**Objective:**

The number of citations to a paper represents the weight of that work in a particular area of interest. Several highly cited papers are listed in the bibliometric analysis. This study aimed to identify and analyze the 100 most cited papers in insomnia research that might appeal to researchers and clinicians.

**Methods:**

We reviewed the Web of Science (WOS) Core Collection database to identify articles from 1985 to 24 March 2022. The R bibliometric package was used to further analyze citation counts, authors, year of publication, source journal, geographical origin, subject, article type, and level of evidence. Word co-occurrence in 100 articles was visualized using VOS viewer software.

**Results:**

A total of 44,654 manuscripts were searched on the Web of Science. Between 2001 and 2021, the top 100 influential manuscripts were published, with a total citation frequency of 38,463. The top countries and institutions contributing to the field were the U.S. and Duke University. Morin C.M. was the most productive author, ranking first in citations. *Sleep* had the highest number of manuscripts published in the top 100 (*n* = 31), followed by *Sleep Medicine Reviews* (*n* = 9). The most cited manuscript (Bastien et al., Sleep Medicine, 2001; 3,384 citations) reported clinical validation of the Insomnia Severity Index (ISI) as a brief screening indicator for insomnia and as an outcome indicator for treatment studies. Co-occurrence analyses suggest that psychiatric disorders combined with insomnia and cognitive behavioral therapy remain future research trends.

**Conclusion:**

This study provides a detailed list of the most cited articles on insomnia. The analysis provides researchers and clinicians with a detailed overview of the most cited papers on insomnia over the past two decades. Notably, COVID-19, anxiety, depression, CBT, and sleep microstructure are potential areas of focus for future research.

## Introduction

Insomnia has been emerging with more public concerns over the past decades for affecting people’s health and well-being worldwide. The prevalence of insomnia disorder is approximately 10–20%, with approximately 50% having a chronic course (1). In America, 27.3% of adults reported insomnia 1 year, and the US annual loss of quality-adjusted life-years associated with insomnia (5.6 million) was significantly larger than that associated with any of the other 18 medical conditions assessed, including arthritis (4.94 million), depression (4.02 million), and hypertension (3.63 million) (2). The economic consequences of the disorder and the cost-effectiveness of insomnia treatments, in aggregate, exceeded $100 billion per year, with the majority being spent on indirect costs such as poorer workplace performance, increased health care utilization, and increased accident risk (3). Insomnia has been a public health issue and an extensive concern for medical practitioners. The number of insomnia-associated studies has gradually increased annually, of which 27,399 were published accumulatively in Web of Science (WoS) Core Collection from 1985 to 2021.

With a trend of research interest and explosive publication, it is worth identifying the most influential scientific achievements from an abundance of literature on insomnia related topics. So far, there is no perfect method for evaluating the scientific impact that a specific study has had on a scientific discipline, the number of citations of an article is a proxy to indicate the importance of the study (4). Bibliometric sciences offer both a statistical and quantitative analysis of published articles and provide a measure of their impact in a particular field of research. To date, no such analyses have been performed exploring the most influential works presented in the field of insomnia. In the present study, we aimed to analyze the top 100 most cited articles over the past decades in the field of insomnia with bibliometric citation analysis.

## Methods

### Identification of the top 100 cited articles

The Clarivate Analytics Web of Science Core Collection database was systematically searched on March 31, 2022. The search terms were “insomnia” and “disorders of initiating and maintaining Sleep,” with publication timespan (1985–2022). The publications were ranked by the number of citations, and these were reviewed to identify the top 100 papers with the most citations. Only original articles and reviews with full manuscripts that focused on insomnia as the main topic were included. Literature reviews that briefly summarized published studies were excluded; editorials and consensus statements were excluded. Two reviewers (SL and JJ) independently identified the top 100 papers according to the total citations of the papers, and any disagreement between the 2 reviewers was resolved by consensus involving a third reviewer (XM).

### Analysis of the top 100 cited articles

Publications were stratified and systematically assessed according to publication year, country or institute, authors, and journal. Additionally, the frequencies of keywords extracted from the articles were assessed and then included in a network analysis of the development of insomnia.

All data were downloaded from the Web of Science and imported into the bibliometric package (Version 3.0.0) in R software (Version 4.1.3) (5), which converts and analyses automatically, including the distribution of countries/regions, years of publication, and authors. Publication quality by author was assessed based upon metrics that included the number of publications, citations in the research area, publication h-index value. The h-index is used to quantify an individual’s scientific research output and measure his citation impact (6).

Networks were constructed using VOS viewer v.1.6.18 (7) (Centre for Science and Technology Studies, Leiden University, Leiden, The Netherlands), which is commonly used to analyze and visualize relationships among authors, countries, co-citations, keywords, and the terms used in articles.

The Shapiro–Wilk test was applied to test the normality of the distribution of individual variables. We show the mean and standard deviation for data with a regularly distributed distribution and the median and range for data with a skewed distribution. The Tukey method was also employed for plotting the whiskers and outliers. The *p*-values from pairwise *t*-tests were adjusted according to either the Bonferroni *post-hoc* test or Mann–Whitney test to correct for the performance of multiple statistical analyses. All *p*-values were two-tailed, and a *p*-value of ≤0.05 was considered to indicate statistical significance. We used a one-way analysis of Kruskal–Wallis test for skewed data. The Mann–Kendall rank correlation was employed to test for correlations among non-parametric variables.

## Results

### Global trends of annual publication

A total of 44,654 eligible publications were listed in peer-reviewed journals on the ISI Web of Knowledge WoS Core on 31 March 2022. Manuscripts were screened according to inclusion and exclusion criteria and ranked according to citation frequency. The top 100 influential manuscripts were obtained. General information is detailed in Table 1.

**TABLE 1:** The top 100 most-cited articles in insomnia.

Rank	References	Journal	Title	Citations	Citations/ Year since publication	Citations in 2021
1	Bastien et al. (9)	Sleep Medicine	Validation of the insomnia severity index as an outcome measure for insomnia research	3,384	153.82	630
2	Lai et al. (8)	JAMA Network Open	Factors associated with mental health outcomes among health care workers exposed to coronavirus disease 2019	2,680	893.33	1,586
3	Ohayon et al. (10)	Sleep Medicine Review	Epidemiology of insomnia: What we know and what we still need to learn	2,126	101.24	179
4	Wittchen et al. (11)	European Neuropsychopharmacology	The size and burden of mental disorders and other disorders of the brain in Europe 2010	2,082	173.5	255
5	Morin et al. (12)	Sleep	The insomnia severity index: Psychometric indicators to detect insomnia cases and evaluate treatment response	1,571	130.92	403
6	Baglioni et al. (13)	Journal of Affective Disorders	Insomnia as a predictor of depression: A meta-analytic evaluation of longitudinal epidemiological studies	1,173	97.75	168
7	Hofmann et al. et al. (14)	Cognitive Therapy and Research	The efficacy of cognitive behavioral therapy: A review of meta-analyses	1,150	104.55	215
8	Pappa et al. (15)	Brain Behavior and Immunity	Prevalence of depression, anxiety, and insomnia among healthcare workers during the COVID-19 pandemic: A systematic review and meta-analysis	1,112	370.67	801
9	Torales et al. (16)	International Journal of Social Psychiatry	The outbreak of COVID-19 coronavirus and its impact on global mental health	1,052	350.67	676
10	Kripke et al. (17)	Archives of General Psychiatry	Mortality associated with sleep duration and insomnia	1,043	49.67	43
11	Sateia (18)	Chest	International classification of sleep disorders-third edition highlights and modifications	923	102.56	321
12	Backhaus et al. (19)	Journal of Psychosomatic Research	Test-retest reliability and validity of the Pittsburgh Sleep Quality Index in primary insomnia	917	43.67	121
13	Schutte-Rodin et al. (20)	Journal of Clinical Sleep Medicine	Clinical guideline for the evaluation and management of chronic insomnia in adults	901	60.07	109
14	Riemann et al. (21)	Sleep Medicine Reviews	The hyperarousal model of insomnia: A review of the concept and its evidence	792	60.92	107
15	Morin et al. (22)	Sleep	Psychological and behavioral treatment of insomnia: Update of the recent evidence (1998–2004)	749	44.06	44
16	Carney et al. (23)	Sleep	The consensus sleep diary: Standardizing prospective sleep self-monitoring	735	66.82	161
17	Rogers et al. (24)	Lancet Psychiatry	Psychiatric and neuropsychiatric presentations associated with severe coronavirus infections: A systematic review and meta-analysis with comparison to the COVID-19 pandemic	733	244.33	478
18	Morgenthaler et al. (25)	Sleep	Practice parameters for the use of actigraphy in the assessment of sleep and sleep disorders: An update for 2007	727	45.44	46
19	Buysse et al. (26)	Sleep	Recommendations for a standard research assessment of insomnia	691	40.65	60
20	Edinger et al. (27)	Sleep	Derivation of research diagnostic criteria for insomnia: Report of an American academy of sleep medicine work group	677	35.63	45
21	Qaseem et al. (28)	Annals of Internal Medicine	Management of chronic insomnia disorder in adults: A clinical practice guideline from the American College of Physicians	662	94.57	169
22	Littner et al. (29)	Sleep	Practice Parameters for clinical use of the multiple sleep latency test and the maintenance of wakefulness test – an American Academy of Sleep Medicine report – standards of practice committee of the American Academy of Sleep Medicine	657	36.5	52
23	Morin et al. (30)	Sleep Medicine	Epidemiology of insomnia: Prevalence, self-help treatments, consultations, and determinants of help-seeking behaviors	654	38.47	55
24	Glass et al. (31)	BMJ-British Medical Journal	Sedative hypnotics in older people with insomnia: Meta-analysis of risks and benefits	632	35.11	43
25	Pandi-Perumal et al. (32)	FEBS Journal	Melatonin – nature’s most versatile biological signal?	621	36.53	42
26	Zhang and Wang (33)	Sleep	Sex differences in insomnia: A meta-analysis	607	35.71	102
27	Gottlieb et al. (34)	Sleep	Association of usual sleep duration with hypertension: The sleep heart health study	604	35.53	28
28	Tsuno et al. (35)	Journal of Clinical Psychiatry	Sleep and depression	603	33.5	66
29	Xiao et al. (36)	Medical Science Monitor	Social Capital and sleep quality in individuals who self-isolated for 14 days during the coronavirus disease 2019 (COVID-19) outbreak in January 2020 in China	581	193.67	323
30	Riemann et al. (37)	Journal of Sleep Research	European guideline for the diagnosis and treatment of insomnia	557	92.83	217
31	Taylor et al. (38)	Sleep	Epidemiology of insomnia, depression, and anxiety	554	30.78	58
32	Tsai et al. (39)	Quality of Life Research	Psychometric evaluation of the Chinese version of the Pittsburgh Sleep Quality Index (QI) in primary insomnia and control subjects	549	30.5	109
33	Smith and Haythornthwaite (40)	Sleep Medicine Reviews	How do sleep disturbance and chronic pain inter-relate? Insights from the longitudinal and cognitive-behavioral clinical trials literature	543	28.58	36
34	Alvaro et al. (41)	Sleep	A systematic review assessing bidirectionality between sleep disturbances, anxiety, and depression	531	53.1	156
35	Ohayon and Roth (42)	Journal of Psychiatry Research	Place of chronic insomnia in the course of depressive and anxiety disorders	527	26.35	37
36	Zhang et al. (43)	Psychotherapy and Psychosomatics	Mental Health and psychosocial problems of medical health workers during the COVID-19 epidemic in China	521	173.67	346
37	Manber et al. (44)	Sleep	Cognitive behavioral therapy for insomnia enhances depression outcome in patients with comorbid major depressive disorder and insomnia	521	34.73	35
38	Vgontzas et al. (45)	Journal of Clinical Endocrinology & Metabolism	Chronic insomnia is associated with nyctohemeral activation of the hypothalamic-pituitary-adrenal axis: Clinical implications	506	23	28
39	Smith et al. (46)	American Journal of Psychiatry	Comparative meta-analysis of pharmacotherapy and behavior therapy for persistent insomnia	500	23.81	19
40	Morgenthaler et al. (47)	Sleep	Practice parameters for the psychological and behavioral treatment of insomnia: An update. An American Academy of Sleep Medicine Report	496	29.18	53
41	Buckley and Schatzberg (48)	Journal of Clinical Endocrinology & Metabolism	Review: On the interactions of the hypothalamic-pituitary-adrenal (HPA) axis and sleep: Normal HPA axis activity and circadian rhythm, exemplary sleep disorders	496	27.56	56
42	Taylor et al. (49)	Sleep	Comorbidity of chronic insomnia with medical problems	471	29.44	54
42	Buysse et al. (50)	Sleep	Prevalence, course, and comorbidity of insomnia and depression in young adults	468	31.2	40
44	Vgontzas et al. (51)	Sleep	Insomnia with objective short sleep duration is associated with a high risk for hypertension	460	32.86	30
45	Bonnet and Arand (52)	Sleep Medicine Reviews	Hyperarousal and insomnia: State of the science	457	35.15	49
46	Savard and Morin (53)	Journal of Clinical Oncology	Insomnia in the context of cancer: A review of a neglected problem	457	20.77	43
47	Marino et al. (54)	Sleep	Measuring sleep: Accuracy, sensitivity, and specificity of wrist actigraphy compared to polysomnography	452	45.2	103
48	Drake et al. (55)	Sleep	Shift work sleep disorder: Prevalence and consequences beyond that of symptomatic day workers	449	23.63	37
49	Johnson et al. (56)	Journal of Psychiatric Research	The association of insomnia with anxiety disorders and depression: Exploration of the direction of risk	435	25.59	49
50	Nofzinger et al. (57)	American Journal of Psychiatry	Functional neuroimaging evidence for hyperarousal in insomnia	431	22.68	26
51	Rossi et al. (58)	Frontiers in Psychiatry	COVID-19 pandemic and lockdown measures impact on mental health among the general population in Italy	424	141.33	320
52	Littner et al. (59)	Sleep	Practice parameters for the role of actigraphy in the study of sleep and circadian rhythms: An update for 2002 – an American academy of sleep medicine report	424	21.2	17
53	Baglioni et al. (60)	Sleep Medicine Reviews	Sleep and emotions: A focus on insomnia	423	32.54	45
54	Morin and Benca (61)	Lancet	Chronic insomnia	422	38.36	56
55	Cajochen et al. (62)	Journal OF Neuroendocrinology	Role of melatonin in the regulation of human circadian rhythms and sleep	420	21	47
56	Morin et al. (63)	JAMA-Journal of The American Medical Association	Cognitive behavioral therapy, singly and combined with medication, for persistent insomnia a randomized controlled trial	414	29.57	43
57	Hao et al. (64)	Brain Behavior and Immunity	Do psychiatric patients experience more psychiatric symptoms during COVID-19 pandemic and lockdown? A case-control study with service and research implications for immunopsychiatry	401	133.67	253
58	Trauer et al. (65)	Annals of Internal Medicine	Cognitive behavioral therapy for chronic insomnia a systematic review and meta-analysis	401	50.13	80
59	Morin et al. (66)	Psychosomatic Medicine	Role of stress, arousal, and coping skills in primary insomnia	401	23.59	44
60	Buysse et al. (1)	JAMA-Journal of The American Medical Association	Insomnia	394	39.4	84
61	Daley et al. (67)	Sleep	The economic burden of insomnia: Direct and indirect costs for individuals with insomnia syndrome, insomnia symptoms, and good sleepers	394	28.14	48
62	Sateia et al. (68)	Journal of Clinical Sleep Medicine	Clinical practice guideline for the pharmacologic treatment of chronic insomnia in adults: An American academy of sleep medicine clinical practice guideline	392	65.33	132
63	National Institutes of Health (69)	Sleep	National institutes of health state of the science conference statement – manifestations and management of chronic insomnia in adults June 13–15, 2005	390	21.67	30
64	Edinger et al. (70)	JAMA-Journal of the American Medical Association	Cognitive behavioral therapy for treatment of chronic primary insomnia – a randomized controlled trial	379	17.23	23
65	Riemann et al. (71)	Journal of Affective Disorders	Primary insomnia: A risk factor to develop depression?	376	18.8	13
66	Neckelmann et al. (72)	Sleep	Chronic insomnia as a risk factor for developing anxiety and depression	373	23.31	36
67	Fortier-Brochu et al. (73)	Sleep Medicine Reviews	Insomnia and daytime cognitive performance: A meta-analysis	364	33.09	53
68	Vgontzas et al. (74)	Sleep Medicine Reviews	Insomnia with objective short sleep duration: The most biologically severe phenotype of the disorder	362	36.2	61
69	Lichstein et al. (75)	Sleep	Actigraphy validation with insomnia	354	20.82	25
70	Morin et al. (76)	Sleep	Dysfunctional beliefs and attitudes about sleep (DBAS): Validation of a brief version (DBAS-16)	350	21.88	44
71	Stepanski and Wyatt (77)	Sleep Medicine Reviews	Use of sleep hygiene in the treatment of insomnia	350	17.5	39
72	Altena et al. (78)	Journal of Sleep Research	Dealing with sleep problems during home confinement due to the COVID-19 outbreak: Practical recommendations from a task force of the European CBT-I Academy	348	116	222
73	Morphy et al. (79)	Sleep	Epidemiology of insomnia: A longitudinal study in a UK population	348	21.75	25
74	Sivertsen et al. (80)	JAMA-Journal of The American Medical Association	Cognitive behavioral therapy vs zopiclone for treatment of chronic primary insomnia in older adults – a randomized controlled trial	342	20.12	23
75	Espie (81)	Annual Review of Psychology	Insomnia: Conceptual issues in the development, persistence, and treatment of sleep disorder in adults	331	15.76	20
76	Johnson et al. (82)	Pediatrics	Epidemiology of DSM-IV insomnia in adolescence: Lifetime prevalence, chronicity, and an emergent gender difference	330	19.41	37
77	Bertolazi et al. (83)	Sleep Medicine	Validation of the Brazilian Portuguese version of the Pittsburgh Sleep Quality Index	323	26.92	72
78	Irwin et al. (84)	Health Psychology	Comparative meta-analysis of behavioral interventions for insomnia and their efficacy in middle-aged adults and in older adults 55+ years of age	322	18.94	16
79	Jacobs et al. (85)	Archives of Internal Medicine	Cognitive behavior therapy and pharmacotherapy for insomnia – a randomized controlled trial and direct comparison	319	16.79	19
80	Soldatos et al. (86)	Journal of Psychosomatic Research	The diagnostic validity of the Athens Insomnia Scale	314	15.7	54
81	Jansson-Frojmark and Lindblom (87)	Journal of Psychosomatic of Research	A bidirectional relationship between anxiety and depression, and insomnia? A prospective study in the general population	313	20.87	45
82	Krystal et al. (88)	Sleep	Sustained efficacy of eszopiclone over 6 months of nightly treatment: Results of a randomized, double-blind, placebo-controlled study in adults with chronic insomnia	311	15.55	11
83	Prober et al. (89)	Journal of Neuroscience	Hypocretin/orexin overexpression induces an insomnia-like phenotype in zebrafish	307	18.06	16
84	Savard et al. (90)	Journal of Clinical Oncology	Randomized study on the efficacy of cognitive-behavioral therapy for insomnia secondary to breast cancer, part I: Sleep and psychological effects	304	16.89	11
85	Youngstedt (91)	Clinics in Sports Medicine	Effects of exercise on sleep	304	16.89	33
86	Fava et al. (92)	Biological Psychiatry	Eszopiclone co-administered with fluoxetine in patients with insomnia coexisting with major depressive disorder	301	17.71	9
87	Vgontzas et al. (93)	Diabetes Care	Insomnia with objective short sleep duration is associated with type 2 diabetes a population-based study	300	21.43	18
88	Morin et al. (94)	Archives of Internal Medicine	The Natural history of insomnia a population-based 3-year longitudinal study	300	21.43	31
89	Perlis et al. (95)	Sleep	Beta/Gamma EEG activity in patients with primary and secondary insomnia and good sleeper controls	295	13.41	21
90	Leger et al. (96)	Sleep	Medical and socio-professional impact of insomnia	291	13.86	8
91	Sofi et al. (97).	European Journal of Preventive Cardiology	Insomnia and risk of cardiovascular disease: A meta-analysis	287	31.89	54
92	Buysse et al. (98)	Archives of Internal Medicine	Efficacy of brief behavioral treatment for chronic insomnia in older adults	286	23.83	27
93	LeBlanc et al. (99)	Sleep	Incidence and risk factors of insomnia in a population-based sample	285	20.36	36
94	Wilson et al. (100)	Journal of Psychopharmacology	British Association for Psychopharmacology consensus statement on evidence-based treatment of insomnia, parasomnias and circadian rhythm disorders	283	21.77	21
95	Palesh et al. (101)	Journal of Clinical Oncology	Prevalence, demographics, and psychological associations of sleep disruption in patients with cancer: University of Rochester cancer center-community clinical oncology program	283	21.77	40
96	Kessler et al. (102)	Sleep	Insomnia and the performance of US workers: Results from the America Insomnia Survey	279	23.25	39
97	Reid et al. (103)	Sleep Medicine	Aerobic exercise improves self-reported sleep and quality of life in older adults with insomnia	276	21.23	34
98	Espie et al. (104)	Sleep	A randomized, placebo-controlled trial of online cognitive behavioral therapy for chronic insomnia disorder delivered *via* an automated media-rich web application	275	22.92	50
99	Harrison and Keating (105)	CNS Drugs	Zolpidem – a review of its use in the management of insomnia	274	15.22	22
100	Zachariae et al. (106)	Sleep Medicine Reviews	Efficacy of internet-delivered cognitive-behavioral therapy for insomnia – a systematic review and meta-analysis of randomized controlled trials	270	38.57	68

**FIGURE 1:**
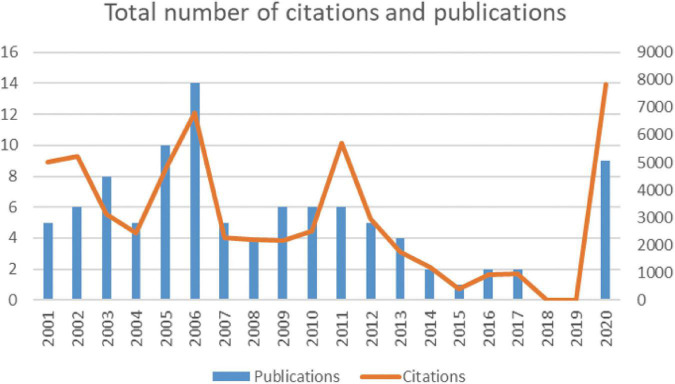
Global trends in publications on insomnia research.

Over the course of 20 years, the total number of citations for the top 100 works of literature varied, but reached a peak in 2021 (Figure 1). The total citation frequency of the top 100 highly cited literature was 58,229 (ranging from 270 to 3,384), with a mean citation frequency of 582.29 and a median citation frequency of 427.5. To exclude the effect of year on citation volume, we analyzed the average annual citation rate of the 100 documents, the highest of which was “Factors Associated With Mental Health Outcomes Among Health Care Workers Exposed to Coronavirus Disease 2019” by Lai et al. (8) (average annual citation rate of 893.33; Table 1).

**FIGURE 2:**
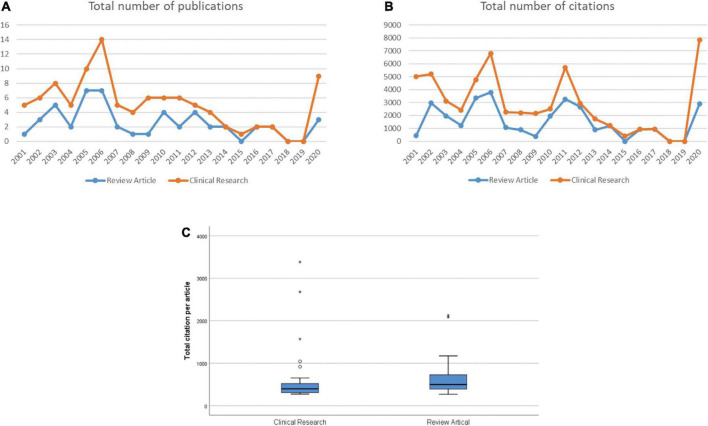
**(A)** The total number of publications for each type of article (clinical or review article) according to publication year. **(B)** The total number of citations publications for each type of article (clinical or review article) according to publication year. **(C)** Bar graph showing the number of citations (and standard deviation) for the 100 most-cited articles according to type of article (clinical research, review article). Box: lower linee= Box: lower linee number of= Box: lower linee number of citati= median value, white points = outliers. The Tukey method was used for plotting the whiskers and outliers. *Outlier.

**FIGURE 3:**
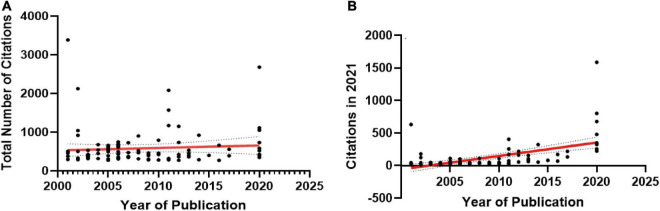
**(A)** Overall citation rate since publication, and **(B)** current (2021 = last full year) citation rate for the 100 most-cited articles according to the publication date of the article.

Most articles on the list were published from 2005 to 2006 (*n* = 24), followed by articles published from 2020 (*n* = 9; Figure 2A). The number of citations was high for articles published between 2001 and 2012 (mean total citations = 3762) and decreased for articles published after 2012, but reached a peak in 20 years for articles published in 2020 (citations = 7852; Figure 2B). The total citation rate of an article was not related to the date of publication (*r* = 0.07108, *p* > 0.05, Mann–Kendall test; Figure 3A). However, the current citation rate of an article (as measured by the number of citations in 2021) suggests that articles published after 2011 are more likely to have been cited in recent years. This correlation was statistically significant (*r* = 0.5394, *p* < 0.0001, Mann–Kendall test: Figure 3B).

Of the 100 articles, 50 were clinical research, 49 were review articles and 1 was basic research. Due to the small sample size of the basic research, we analyzed the number of citations for review articles and clinical research (Table 2) and found that the review articles did not vary significantly with respect to total citations per article compared to the clinical research articles [Mann–Whitney test, *p* = 0.08; clinical research: median = 397.5 (range = 275–3384); review articles: median = 531(range = 270–2126): Figure 2C].

**TABLE 2:** Citations for review articles and clinical research.

	Citations for review articles and clinical research				*P* *
	***N***	**Range (min/max)**	**Mean (±SD)**	**Median (Q25/Q75)**	**Gr2 vs Gr3**
Clinical research (Gr2)	50	275/3,384	546.86 (557.47)	397.5 (311.5/517.25)	*P* = 0.05
Review article (Gr3)	49	270/2,126	624.06 (384.40)	500 (390/727)	*Mann–Whitney test

*P ≤ 0.05.

### Distribution of countries and institutes

**FIGURE 4:**
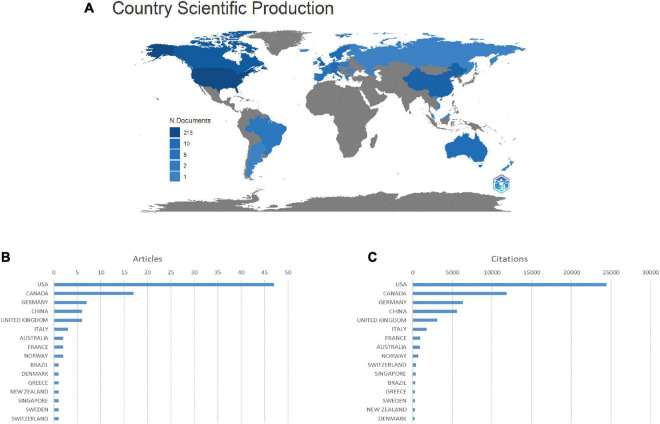
Countries contributing to insomnia research. **(A)** World map showing the distribution of countries in this field. **(B)** Top 15 countries with the largest number of publications. **(C)** Total citations of related articles from different countries.

The global contribution of insomnia research was analyzed and represented by a blue-coded world map in the R software (Figure 4A). Of the 35 countries and territories identified for this study, the USA had the highest number of articles (*n* = 56), followed by Canada (*n* = 22), Germany (*n* = 11), Italy (*n* = 10) and the UK (*n* = 7) (Figure 4B). Studies from the USA were the most cited (24,423 citations), followed by Canada (11,832 citations), Germany (6,329 citations), China (5,587 citations) and the UK (3,097 citations) (Figure 4C).

**FIGURE 5:**
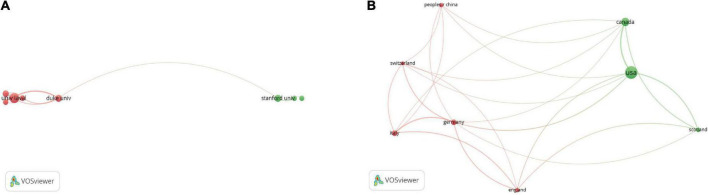
Co-authorship analysis of countries and institutions. **(A)** Network map of co-authorship between countries with more than five publications. **(B)** Network map of co-authorship between institutions with more than five publications. The thickness of the lines indicates the strength of the relationship.

In the co-authorship analysis, a total of eight countries with more than five publications in the field were analyzed (Figure 5A). The five countries with the highest total connection intensity were the United States (total link strength = 19 times), Germany (17 times) and Canada (15 times). A total of 235 institutions are involved in this field. Laval University (38 articles) contributed the most publications, followed by Harvard University (11 articles), Stanford University (10 articles), University of Pittsburgh (10 articles), and Duke University (9 articles). We analyzed the co-authorship of 235 institutions with more than five publications. Eight institutional collaborations are shown (Figure 5B). The strongest institutions overall were Duke University (total link strength = 14 times).

### Analysis of author

**FIGURE 6:**
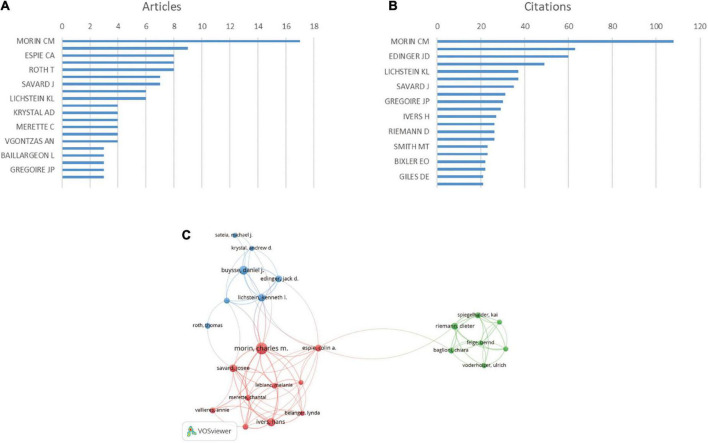
Analysis of authors. **(A)** Number of publications from different authors. **(B)** Total citations in the research filed from different authors. **(C)** Network map of co-authorship between authors with more than five publications. Size of the circles indicate the number of articles in the 100 most cited list, while the width of the curved line represents the link strength. The distance between two authors indicates approximate relatedness among the nodes.

Considering the number of publications, MORIN CM. is the most productive author, with 17 articles (Figure 6A) MORIN CM. was also the top-ranked author in terms of citations in this field (108 citations) (Figure 6B).

We analyzed a total of 481 authors, 60 of whom were co-authors in more than two publications. Excluding 36 unrelated items, 24 authors were shown to have collaborated (Figure 6C). The author with the highest total linkage intensity was MORIN CM. (total link strength = 40 times).

### Analysis of most cited journal

**FIGURE 7:**
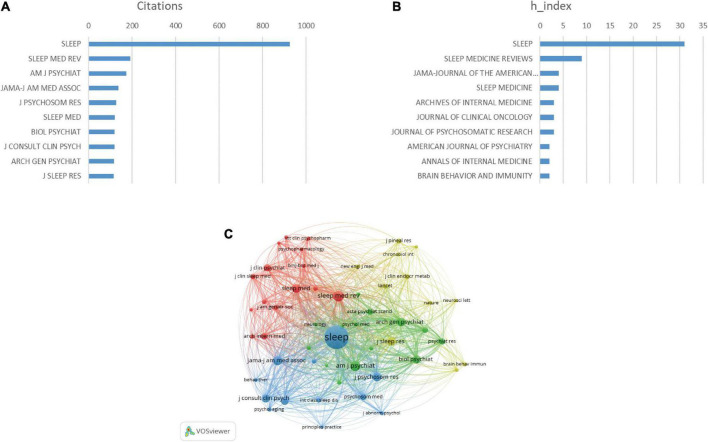
Analysis of journals. **(A)** Total citations in the research filed from different journals. **(B)** h-index of publications from different journals. **(C)** Network map of journals that were co-cited in more than 50 publications. The size of the circle represents the number of papers in the top 100 list.

The 100 articles were published in 42 journals. Figure 7 shows the top ten h-index and cited journals that published related articles (Figures 7A, B). Of these 42 journals, the highest h-index was *Sleep* (h-index = 31), followed closely by *Sleep Medicine Reviews* (h-index = 9). *Sleep* was cited the most (928 times), followed by *Sleep Medicine Reviews* (193 times). In the co-citation analysis, we analyzed a total of 1,352 journals, and a total of 52 journals were cited more than 20 times (Figure 7C).

### Co-occurrence analysis of keywords

**FIGURE 8:**
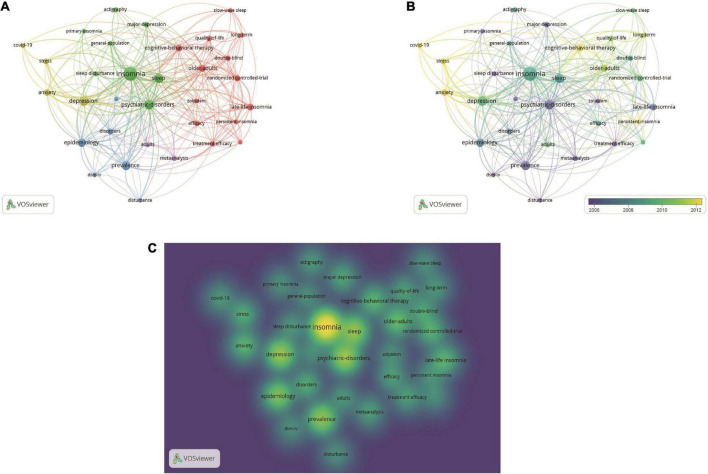
Co-occurrence analysis of keywords. **(A)** Mapping of keywords of studies. **(B)** Distribution of keywords according to average publication year (blue: earlier, yellow: later). **(C)** Distribution of keywords according to the mean frequency of appearance. Keywords in yellow occurred with the highest frequency.

We analyzed a total of 33 keywords that were identified as appearing more than five times (Figure 8A). The colors in the overlay visualization shown in Figure 8B indicate the average year of publication of the identified keywords. The keywords which published after 2011 are colored more green or yellow. The density visualization shows the same identified keywords mapped by frequency of occurrence (Figure 8C).

### Citation and co-citation analyses

**FIGURE 9:**
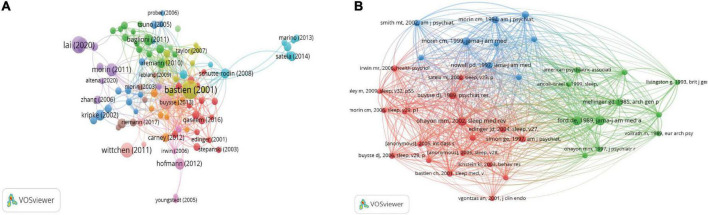
**(A)** Network map of citation analysis of documents with more than 50 citations. **(B)** Network map of co-citation analysis of references with more than 10 citations. The size of the circle represents the number of papers in the top 100 list.

The citation analysis showed 94 pieces of literature with more than 50 citations (Figure 9A). As shown in Table 1, “Validation of the Insomnia Severity Index as an outcome measure for insomnia research” [Bastien et al. (8)] was cited 3,384 times, followed by “Factors Associated With Mental Health Outcomes Among Health Care Workers Exposed to Coronavirus Disease 2019” [Lai et al. (8)] with 2,680 citations and the third most cited is “Epidemiology of insomnia: what we know and what we still need to learn” [Ohayon (10)], with 2,126 citations.

We analyzed 38 references that were co-cited more than 10 times in total (Figure 9B). The three most cited references were Ford de. (107) (1989, JAMA-J AM MED ASSOC; 33 citations), Ohayon (10) mm. (2002, sleep med rev; 30 citations), and Breslau (108) (1996, bio psychiat; 29 citations).

## Discussion

### General trends in insomnia research

Bibliometrics allows for quantitative analysis of a researcher’s individual achievements, or even a country’s or institution’s contribution and international impact in the field, through a statistical analysis of the total number of academic papers published in a clinical field and the total frequency of citations (109). In this study, we combined bibliometric analysis with network visualization to identify the initial 100 most influential manuscripts in the field of insomnia based on global citation frequency, highlighting the contributions that have led to significant advances in insomnia research and pointing to current trends in the field.

With the largest number of publications and citations, and the highest co-authorship analysis ranking by country, the United States is currently the world leader in insomnia research. These results suggest that the US is likely to have a major impact on the direction of research in the field and has the strongest collaboration globally. The citations of articles from Canada, Germany, Italy and the UK have also increased significantly over the last three decades. China has a small total number of publications, but ranks fourth in total citations; it ranks sixth in collaborations with other countries, indicating that China has an influential publication in the field of insomnia and actively maintains close collaborations with other countries. The Laval University Institute is the most productive, with 38% of the publications, while Duke University ranks first in the co-authorship analysis, indicating its close cooperation with other institutes.

### Influential authors and studies in insomnia

Morin, C.M. has the largest number of publications and citations and also ranks first in co-authorship analysis conducted by authors. Of these top 100 highly cited publications, Morin C.M. has published 17 articles, 8 of which he is the first author. Dr. Morin is interested in the validation of assessment scale for insomnia. Utilizing scales to evaluate the therapeutic effects of insomnia is the most convenient and widely used method, and the reliability and validity analysis results are critical for the scale to be used as an outcome indicator. Dr. Morin examined psychometric indices of the Insomnia Severity Index to evaluate treatment response in a clinical sample (9), and validated the Dysfunctional beliefs and attitudes about sleep, providing a variety of indicators for the assessment of insomnia (10). Nevertheless, the original version of the ISI and the PSQI are the most commonly used, and the original version of the ISI remains the only validated scale that is highly recommended for all insomnia research protocols. Dr. Morin has also conducted extensive research on cognitive behavioral therapy for insomnia and its comorbidities (110, 111). His team’s current efforts continue to focus on evaluating the efficacy of cognitive behavioral therapy and optimizing the procedural approach (112, 113).

According to the literature citation analysis and reference co-citation analysis, the most frequently cited Bastien et al. (8) reported clinical validation of the Insomnia Severity Index Scale (ISI) as a brief screening indicator for insomnia and an outcome indicator for treatment studies, indicating that the ISI is a reliable and valid tool for quantifying perceived insomnia severity. The ISI is a brief self-report instrument designed to assess subjective symptoms and daytime status of insomnia and the extent to which insomnia causes worry or distress. The ISI has been continually validated and the study by Bastien et al. was the first formal psychometric analysis of the reliability and validity of the ISI. Further validation of the ISI using item response theory (IRT) analysis was reported by Morin et al. (114), obtaining evidence on internal consistency, item response patterns and convergent validity, yielding new evidence on optimal sensitivity and specificity indices for case finding and assessment of minimal important changes following treatment (12). A recent meta-analysis (115) reported on the construct validity of the Insomnia Severity Index (ISI), which showed that studies reporting validated factor analyses (CFA) had more reliable results than those reporting only exploratory factor analyses (EFA), and that two-factor solution were strong expressions of dimensionality and higher reliability indicators for the ISI compared to three-factor solutions.

### Future outlook

Our co-occurrence network diagram, categorized by subject area or date of publication, shows current hotspots and future directions in insomnia research (Figures 8A–C). The keywords indicate that insomnia research involves a wide range of populations (elderly, adolescents), causal factors (quality of life, coronavirus), disorders (anxiety disorders, depression) and therapies (cognitive behavioral therapy, pharmacotherapy). The most recent keywords indicating future trends in the field are as follows:

### COVID-19 and insomnia

The total number of citations in the insomnia-related literature rose significantly in 2021, reaching a peak in the last 20 years. This may be related to the outbreak of the Corona Virus Disease 2019 (COVID-19). The impact of the new coronavirus pneumonia outbreak has led to an increase in psychological disorders in the population and a climb in the prevalence of insomnia, with 36.7% of adults from 13 countries having clinical symptoms of insomnia and 17.4% meeting diagnostic criteria for insomnia (24, 114). Spielman identified negative life events and other stressors as triggers for the development of insomnia (116), with up to 37% of the population experiencing insomnia in the presence of stressful events (117). During the early stage of the COVID-19 pandemic, insomnia symptoms were mainly associated with acute psychological reactions due to the rapid spread of the disease and strict enforcement of restrictions, as well as poor sleep hygiene (118). During the late stage, insomnia symptoms are associated with economic stress associated with the COVID-19 pandemic (118) and the impairment of sleep patterns (119). Recent studies have shown that into the late stages, sleep is characterized by significant objective sleep fragmentation in the presence of adequate sleep duration (120), suggesting that the adverse effects of the initial pandemic outbreak on sleep will persist. Studies of insomnia during the COVID-19 pandemic highlight the importance of focusing not only on the primary diseases, but also on the psycho-psychological issues, particularly insomnia during global public health events.

### Depression, anxiety, and insomnia

In our analysis of keywords, we found that “anxiety,” “depression,” and “mental disorders” were frequently mentioned in 100 documents as the second most frequently occurring keywords after “insomnia.” Studies have shown that there is a strong relationship between insomnia, depression and anxiety, with insomnia considered a risk factor for anxiety and depression (121), with those suffering from insomnia 9.82 times more likely to have clinically significant depression and 17.35 times more likely to have clinically significant anxiety compared to those without insomnia (38). Anxiety and depression are also considered risk factors for insomnia (122), suggesting that insomnia is bilaterally associated with psychiatric disorders such as anxiety and depression (41). In terms of biological mechanisms, polymorphisms and dysregulation of the serotonin, dopamine(DA), oxytocin (OXT) and genes may be associated with the development and maintenance of insomnia and mood disorders (123), while behavior and thoughts can in turn affect the activity of the serotonin, DA, OXT, and genes (124). In terms of brain function, sleep disturbances have been shown to disrupt the function of cortical neural circuits, including the amygdala, striatum, anterior cingulate cortex and prefrontal cortex (PFC) (125), which play a key role in the regulation of the affective system (126). In addition, there is growing evidence that insomnia disrupts brain functions associated with the reward system (127, 128), and that dysfunction of the reward system is associated with a variety of neuropsychiatric disorders (129), including depression, bipolar disorder (127, 128, 130) and others.

### Subtypes of insomnia

Insomnia is a heterogeneous disorder (131), and identifying clinically relevant subtypes of insomnia disorders can help reduce heterogeneity, identify etiology, and personalize treatment (132). In Ohayon (10) proposed that epidemiological studies should focus on distinguishing different subtypes of insomnia. Typing by sleep stage symptoms, such as difficulty falling asleep (DIS), difficulty maintaining sleep (DMS), early awakening (EMA), or a combination of four subtypes (133); typing by insomnia episodes and duration, such as chronic insomnia, short-term insomnia (134); and typing by primary and secondary clinical features of insomnia, such as primary insomnia, secondary insomnia (135). Although these subtypes can differ in terms of stable sleep-related characteristics, reliability and validity are lacking and heterogeneity still prevails (136). It remains difficult to find consistent insomnia features in terms of cognition, mood, personality, life history, polysomnography, and sleep microstructure, and this inconsistency suggests that different subtypes of insomnia disorder have not been fully identified (137). For a long time, researchers have been working on different aspects of the subtypes of insomnia disorders, such as natural history of insomnia (94), subjective and objective sleep duration (74), sleep microstructure (138), non-insomnia characteristics (life history, affective and personality traits) (137), and clustering subtypes of insomnia (subtyping based on subjective sleep variables as well as age at onset of insomnia, the severity of anxiety and depressive symptoms) (139). Vgontzas et al. (74) proposed that insomnia with short objective sleep duration is the most biologically severe phenotype of the disorder and is associated with a higher risk for hypertension, diabetes, and other diseases. Also, it appears that insomnia with objective short sleep duration is a biological marker of genetic predisposition to chronic insomnia. In the future, the underlying genetic, neurobiological, and neuropsychological mechanisms of insomnia with objective short sleep duration could be further explored. In terms of polysomnography (140), brain imaging (141), and genetics (142), we can also examine the association of other sleep variables with other phenotypes of insomnia.

### Cognitive behavioral therapy

Cognitive behavioral therapy (CBT) is the most widely researched form of psychotherapy, which leads to changes in emotional distress and problem behavior by altering therapeutic strategies that are maladaptive to poor cognition (14). CBT for insomnia (CBT-I) has long been shown to be more effective than control therapy (143). Cognitive behavioral therapy for insomnia (CBT-I) is now commonly recommended as a first-line treatment for chronic insomnia because of the potential for sustained benefit from psychotherapy without the risk of tolerance or adverse effects associated with pharmacological approaches (65). Recent evidence suggests that CBTI can also be used to treat acute insomnia caused by stress (144). Many elements of this treatment approach can be applied to stressful events such as the current COVID-19 pandemic and can be adapted to treat and prevent sleep problems resulting from confinement, increased stress and changes in circadian and daily activities (78). The development of technologies such as the Internet, big data and artificial intelligence has brought about a boom in digital medicine in the healthcare industry, enabling the digitization of CBT-I (145), and the effectiveness of digital cognitive behavioral therapy (dBT-I) for insomnia has been validated (146). In recent years, dBT-I has been widely used during the COVID-19 pandemic, and Liu et al’s (147) study provides an entry point for building a dBTI platform and a theoretical basis for clinical application.

### Sleep microstructure

In recent years, sleep microstructure has gradually gained widespread attention, and a number of the 100 articles we examined have begun to focus on slow-wave sleep. Slow wave sleep (SWS) is a component of non-REM sleep that is important in neurophysiological processes like memory and cognition (148). According to the “active system consolidation hypothesis,” slow oscillations, in conjunction with sleep spindle waves, drive the repetitive reactivation of newly encoded memories during slow-wave sleep, facilitating their integration into long-term memory storage sites (149). A growing body of research confirms that auditory stimulation (150), transcranial direct current stimulation (151), and medication (152) are all effective in improving memory function by enhancing slow waves of sleep (153). Slow-wave sleep is not only used for memory enhancement, but has also been widely used to improve cognitive function in patients with mental illness (154, 155) and for sensory-motor recovery in stroke patients (156). Recent studies have shown that enhancing SWS in healthy individuals profoundly affects the connections between the endocrine and autonomic nervous systems (157), opening up a wide range of potential applications for enhancing SWS.

### Strengths and limitations

To the best of our knowledge, this is the first bibliometric analysis of the Insomnia research trend. Using the R bibliometric package, we conducted a comprehensive survey of the literature to perform quantitative and qualitative analyses of the publication output and quality of studies from various authors. We also used a well-known scientometric software tool (VOSviewer) to build and visualize the bibliometric networks by analyzing co-authorship, co-citation, and co-occurrence. Nevertheless, our analyses have some limitations. Firstly, the search is primarily conducted in the WoS database. Although WoS is the most commonly used database in scientometrics, it is advisable to combine the results with those from other databases, such as PubMed and Scopus. Secondly, our search did not separate mechanistic studies from clinical studies, ignoring the research progress in mechanistic studies; however, this could also indicate that mechanistic studies in the field of sleep could be strengthened. Third, the keyword analysis results may have been influenced by incomplete keyword extraction. To better display the keywords, keywords that appeared more than five times in the network were shown. Fourthly, as this is a developing area of research, we may have overlooked the contribution of analyzing recently published studies because of their low citation frequency, despite some studies being published in high quality journals.

## Conclusion

In conclusion, to our knowledge, this is the first bibliometric study to identify the 100 most cited papers in insomnia research. Our results suggest that the outbreak of the COVID-19 epidemic is strongly associated with the onset of insomnia and stimulates the researcher’s interest. The key words suggest “COVID-19;” “anxiety,” “depression,” “CBT,” and “sleep microstructure” are currently hot topics in the field of insomnia and will be future research trends in the field, indicating that the focus of research has shifted from insomnia epidemiology and scale validation to the study of co-morbidities and sleep microstructure of insomnia. Despite its limitations, citation analysis provides an important quantitative approach to research in the field of comparative science. The findings of this study may provide a valuable reference for researchers to guide and implement their scientific research interests in the field of insomnia.

## Author contributions

QW, KL, and WW designed the study. QW and KL wrote and revised the draft manuscript and carried out data visualization and graphical interpretation. QW, KL, SL, JJ, and XW performed the literature search, retrieval, and data collection. WW provided the critical assistance or funding. All authors contributed and approved the final draft of the manuscript before submission.

## References

[1] Buysse DJ. Insomnia. *JAMA.* (2013) 309:706–16. 10.1001/jama.2013.193 23423416 PMC3632369

[2] Olfson M Wall M Liu S Morin C Blanco C. Insomnia and impaired quality of life in the United States. *J Clin Psychiatry.* (2018) 79:17m12020. 10.4088/JCP.17m12020 30256547

[3] Wickwire E Shaya F Scharf S. Health economics of insomnia treatments: the return on investment for a good night’s sleep. *Sleep Med Rev.* (2016) 30:72–82. 10.1016/j.smrv.2015.11.004 26874067

[4] Landreneau J Weaver M Delaney C Aminian A Dimick J Lillemoe K The 100 most cited papers in the history of the American surgical association. *Ann Surg.* (2020) 271:663–70. 10.1097/SLA.0000000000003633 31663970

[5] Hirsch JE. An index to quantify an individual’s scientific research output. *Proc Natl Acad Sci USA.* (2005) 102:16569–72. 10.1073/pnas.0507655102 16275915 PMC1283832

[6] Aria M Cuccurullo C. bibliometric: an R-tool for comprehensive science mapping analysis. *J Informetr.* (2018) 11:959–75. 10.1016/j.joi.2017.08.007

[7] van Eck N Waltman L. Citation-based clustering of publications using CitNetExplorer and VOSviewer. *Scientometrics.* (2017) 111:1053–70. 10.1007/s11192-017-2300-7 28490825 PMC5400793

[8] Lai J Ma S Wang Y Cai Z Hu J Wei N Factors associated with mental health outcomes among health care workers exposed to coronavirus disease 2019. *JAMA Netw Open.* (2020) 3:e203976. 10.1001/jamanetworkopen.2020.3976 32202646 PMC7090843

[9] Bastien CH Vallières A Morin CM. Validation of the insomnia severity index as an outcome measure for insomnia research. *Sleep Med.* (2001) 2:297–307. 10.1016/S1389-9457(00)00065-4 11438246

[10] Ohayon MM. Epidemiology of insomnia: what we know and what we still need to learn. *Sleep Med Rev.* (2002) 6:97–111. 10.1053/smrv.2002.0186 12531146

[11] Wittchen H Jacobi F Rehm J Gustavsson A Svensson M Jönsson B The size and burden of mental disorders and other disorders of the brain in Europe 2010. *Eur Neuropsychopharmacol.* (2011) 21:655–79. 10.1016/j.euroneuro.2011.07.018 21896369

[12] Morin CM Belleville G Bélanger L Ivers H. The insomnia severity index: psychometric indicators to detect insomnia cases and evaluate treatment response. *Sleep.* (2011) 34:601–8. 10.1093/sleep/34.5.601 21532953 PMC3079939

[13] Baglioni C Battagliese G Feige B Spiegelhalder K Nissen C Voderholzer U Insomnia as a predictor of depression: A meta-analytic evaluation of longitudinal epidemiological studies. *J Affect Disord.* (2011) 135:10–9. 10.1016/j.jad.2011.01.011 21300408

[14] Hofmann SG Asnaani A Vonk IJ Sawyer AT Fang A. The efficacy of cognitive behavioral therapy: a review of meta-analyses. *Cogn Ther Res.* (2012) 36:427–40. 10.1007/s10608-012-9476-1 23459093 PMC3584580

[15] Pappa S Ntella V Giannakas T Giannakoulis V Papoutsi E Katsaounou P. Prevalence of depression, anxiety, and insomnia among healthcare workers during the COVID-19 pandemic: A systematic review and meta-analysis. *Brain Behav Immun.* (2020) 88:901–7. 10.1016/j.bbi.2020.05.026 32437915 PMC7206431

[16] Torales J O’Higgins M Castaldelli-Maia J Ventriglio A. The outbreak of COVID-19 coronavirus and its impact on global mental health. *Int J Soc Psychiatry.* (2020) 66:317–20. 10.1177/0020764020915212 32233719

[17] Kripke D Garfinkel L Wingard D Klauber M Marler M. Mortality associated with sleep duration and insomnia. *Arch Gen Psychiatry.* (2002) 59:131–6. 10.1001/archpsyc.59.2.131 11825133

[18] Sateia M. International classification of sleep disorders-third edition highlights and modifications. *Chest.* (2014) 146:1387–94. 10.1378/chest.14-0970 25367475

[19] Backhaus J Junghanns K Broocks A Riemann D Hohagen F. Test-retest reliability and validity of the Pittsburgh sleep quality index in primary insomnia. *J Psychosom Res.* (2002) 53:737–40. 10.1016/S0022-3999(02)00330-6 12217446

[20] Schutte-Rodin S Broch L Buysse D Dorsey C Sateia M. Clinical guideline for the evaluation and management of chronic insomnia in adults. *J Clin Sleep Med.* (2008) 4:487–504. 10.5664/jcsm.27286 18853708 PMC2576317

[21] Riemann D Spiegelhalder K Feige B Voderholzer U Berger M Perlis M The hyperarousal model of insomnia: a review of the concept and its evidence. *Sleep Med Rev.* (2010) 14:19–31. 10.1016/j.smrv.2009.04.002 19481481

[22] Morin C Bootzin R Buysse D Edinger J Espie C Lichstein K. Psychological and behavioral treatment of insomnia: update of the recent evidence (1998-2004). *Sleep.* (2006) 29:1398–414. 10.1093/sleep/29.11.1398 17162986

[23] Carney C Buysse D Ancoli-Israel S Edinger J Krystal A Lichstein K The consensus sleep diary: standardizing prospective sleep self-monitoring. *Sleep.* (2012) 5:287–302. 10.5665/sleep.1642 22294820 PMC3250369

[24] Rogers JP Chesney E Oliver D Pollak TA McGuire P Fusar-Poli P Psychiatric and neuropsychiatric presentations associated with severe coronavirus infections: a systematic review and meta-analysis with comparison to the COVID-19 pandemic. *Lancet Psychiatry.* (2020) 7:611–27. 10.1016/S2215-0366(20)30203-0 32437679 PMC7234781

[25] Morgenthaler T Alessi C Friedman L Owens J Kapur V Boehlecke B Practice parameters for the use of actigraphy in the assessment of sleep and sleep disorders: an update for 2007. *Sleep.* (2007) 30:519–29. 10.1093/sleep/30.4.519 17520797

[26] Buysse D Ancoli-Israel S Edinger J Lichstein K Morin C. Recommendations for a standard research assessment of insomnia. *Sleep.* (2006) 29:1155–73. 10.1093/sleep/29.9.1155 17040003

[27] Edinger J Bonnet M Bootzin R Doghramji K Dorsey C Espie C Derivation of research diagnostic criteria for insomnia: report of an american academy of sleep medicine work group. *Sleep.* (2004) 27:1567–96. 10.1093/sleep/27.8.1567 15683149

[28] Qaseem A Kansagara D Forciea M Cooke M Denberg T. Management of chronic insomnia disorder in adults: a clinical practice guideline from the American college of physicians. *Ann Intern Med.* (2016) 165:125–33. 10.7326/M15-2175 27136449

[29] Littner M Kushida C Wise M Davila D Morgenthaler T Lee-Chiong T Practice parameters for clinical use of the multiple sleep latency test and the maintenance of wakefulness test–an American academy of sleep medicine report–standards of practice committee of the american academy of sleep medicine. *Sleep.* (2005) 28:113–21. 10.1093/sleep/28.1.113 15700727

[30] Morin C LeBlanc M Daley M Gregoire J Mérette C. Epidemiology of insomnia: prevalence, self-help treatments, consultations, and determinants of help-seeking behaviors. *Sleep Med.* (2006) 7:123–30. 10.1016/j.sleep.2005.08.008 16459140

[31] Glass J Lanctôt K Herrmann N Sproule B Busto U. Sedative hypnotics in older people with insomnia: meta-analysis of risks and benefits. *BMJ.* (2005) 331:1169. 10.1136/bmj.38623.768588.47 16284208 PMC1285093

[32] Pandi-Perumal S Srinivasan V Maestroni G Cardinali D Poeggeler B Hardeland R. Melatonin–nature’s most versatile biological signal? *FEBS J.* (2006) 273:2813–38. 10.1111/j.1742-4658.2006.05322.x 16817850

[33] Zhang B Wing Y. Sex differences in insomnia: a meta-analysis. *Sleep.* (2006) 29:85–93. 10.1093/sleep/29.1.85 16453985

[34] Gottlieb D Redline S Nieto F Baldwin C Newman A Resnick H Association of usual sleep duration with hypertension: the sleep heart health study. *Sleep.* (2006) 29:1009–14. 10.1093/sleep/29.8.1009 16944668

[35] Tsuno N Besset A Ritchie K. Sleep and depression. *J Clin Psychiatry.* (2005) 66:1254–69. 10.4088/JCP.v66n1008 16259539

[36] Xiao H Zhang Y Kong D Li S Yang N. Social capital and sleep quality in individuals who self-isolated for 14 days during the coronavirus disease 2019 (COVID-19) outbreak in january 2020 in China. *Med Sci Monit.* (2020) 26:e923921. 10.12659/MSM.923921 32194290 PMC7111105

[37] Riemann D Baglioni C Bassetti C Bjorvatn B Dolenc Groselj L Ellis J European guideline for the diagnosis and treatment of insomnia. *J Sleep Res.* (2017) 26:675–700. 10.1111/jsr.12594 28875581

[38] Taylor DJ Lichstein KL Durrence HH Reidel B Bush A. Epidemiology of insomnia, depression, and anxiety. *Sleep.* (2005) 28:1457–64. 10.1093/sleep/28.11.1457 16335332

[39] Tsai P Wang S Wang M Su C Yang T Huang C Psychometric evaluation of the Chinese version of the Pittsburgh sleep quality index (CPSQI) in primary insomnia and control subjects. *Qual Life Res.* (2005) 14:1943–52. 10.1007/s11136-005-4346-x 16155782

[40] Smith M Haythornthwaite J. How do sleep disturbance and chronic pain inter-relate? Insights from the longitudinal and cognitive-behavioral clinical trials literature. *Sleep Med Rev.* (2004) 8:119–32. 10.1016/S1087-0792(03)00044-3 15033151

[41] Alvaro P Roberts R Harris JKA. Systematic review assessing bidirectionality between sleep disturbances, anxiety, and depression. *Sleep.* (2013) 36:1059–68. 10.5665/sleep.2810 23814343 PMC3669059

[42] Ohayon M Roth T. Place of chronic insomnia in the course of depressive and anxiety disorders. *J Psychiatry Res.* (2003) 37:9–15. 10.1016/S0022-3956(02)00052-3 12482465

[43] Zhang W Wang K Yin L Zhao W Xue Q Peng M Mental health and psychosocial problems of medical health workers during the COVID-19 epidemic in China. *Psychother Psychosom.* (2020) 89:242–50. 10.1159/000507639 32272480 PMC7206349

[44] Manber R Edinger J Gress J San Pedro-Salcedo M Kuo T Kalista T. Cognitive behavioral therapy for insomnia enhances depression outcome in patients with comorbid major depressive disorder and insomnia. *Sleep.* (2008) 31:489–95. 10.1093/sleep/31.4.489 18457236 PMC2279754

[45] Vgontzas A Bixler E Lin H Prolo P Mastorakos G Vela-Bueno A Chronic insomnia is associated with nyctohemeral activation of the hypothalamic-pituitary-adrenal axis: Clinical implications. *J Clin Endocrinol Metab.* (2001) 86:3787–94. 10.1210/jcem.86.8.7778 11502812

[46] Smith M Perlis M Park A Smith M Pennington J Giles D Comparative meta-analysis of pharmacotherapy and behavior therapy for persistent insomnia. *Am J Psychiatry.* (2002) 159:5–11. 10.1176/appi.ajp.159.1.5 11772681

[47] Morgenthaler T Kramer M Alessi C Friedman L Boehlecke B Brown T Practice parameters for the psychological and behavioral treatment of insomnia: an update. An American academy of sleep medicine report. *Sleep.* (2006) 29:1415–9. 10.1093/sleep/29.11.1415 17162987

[48] Buckley T Schatzberg A. Review: On the interactions of the hypothalamic-pituitary-adrenal (HPA) axis and sleep: Normal HPA axis activity and circadian rhythm, exemplary sleep disorders. *J Clin Endocrinol Metab.* (2005) 90:3106–14. 10.1210/jc.2004-1056 15728214

[49] Taylor D Mallory L Lichstein K Durrence H Riedel B Bush A. Comorbidity of chronic insomnia with medical problems. *Sleep.* (2007) 30:213–8. 10.1093/sleep/30.2.213 17326547

[50] Buysse D Angst J Gamma A Ajdacic V Eich D Rössler W. Prevalence, course, and comorbidity of insomnia and depression in young adults. *Sleep.* (2008) 31:473–80. 10.1093/sleep/31.4.473 18457234 PMC2279748

[51] Vgontzas A Liao D Bixler E Chrousos G Vela-Bueno A. Insomnia with objective short sleep duration is associated with a high risk for hypertension. *Sleep.* (2009) 32:491–7. 10.1093/sleep/32.4.491 19413143 PMC2663863

[52] Bonnet M Arand D. Hyperarousal and insomnia: State of the science. *Sleep Medicine Reviews.* (2010) 14:9–15. 10.1016/j.smrv.2009.05.002 19640748

[53] Savard J Morin C. Insomnia in the context of cancer: a review of a neglected problem. *Journal of Clinical Oncology.* (2001) 19:895–908. 10.1200/JCO.2001.19.3.895 11157043

[54] Marino M Li Y Rueschman M Winkelman J Ellenbogen J Solet J Measuring sleep: accuracy, sensitivity, and specificity of wrist actigraphy compared to polysomnography. *Sleep.* (2013) 36:1747–55. 10.5665/sleep.3142 24179309 PMC3792393

[55] Drake C Roehrs T Richardson G Walsh J Roth T. Shift work sleep disorder: Prevalence and consequences beyond that of symptomatic day workers. *Sleep.* (2004) 27:1453–62. 10.1093/sleep/27.8.1453 15683134

[56] Johnson E Roth T Breslau N. The association of insomnia with anxiety disorders and depression: Exploration of the direction of risk. *J Psychiatr Res.* (2006) 40:700–8. 10.1016/j.jpsychires.2006.07.008 16978649

[57] Nofzinger E Buysse D Germain A Price J Miewald J Kupfer D. Functional neuroimaging evidence for hyperarousal in insomnia. *Am J Psychiatry.* (2004) 161:2126–8. 10.1176/appi.ajp.161.11.2126 15514418

[58] Rossi R Socci V Talevi D Mensi S Niolu C Pacitti F COVID-19 pandemic and lockdown measures impact on mental health among the general population in Italy. *Front Psychiatry.* (2020) 11:790. 10.3389/fpsyt.2020.00790 32848952 PMC7426501

[59] Littner M Kushida C Anderson W Bailey D Berry R Davila D Practice parameters for the role of actigraphy in the study of sleep and circadian rhythms: an update for 2002–an American academy of sleep medicine report. *Sleep.* (2003) 26:337–41. 10.1093/sleep/26.3.337 12749556

[60] Baglioni C Spiegelhalder K Lombardo C Riemann D. Sleep and emotions: a focus on insomnia. *Sleep Med Rev.* (2010) 14:227–38. 10.1016/j.smrv.2009.10.007 20137989

[61] Morin C Benca R. Chronic insomnia. *Lancet.* (2012) 379:1129–41. 10.1016/S0140-6736(11)60750-2 22265700

[62] Cajochen C Kräuchi K Wirz-Justice A. Role of melatonin in the regulation of human circadian rhythms and sleep. *J Neuroendocrinol.* (2003) 15:432–7. 10.1046/j.1365-2826.2003.00989.x 12622846

[63] Morin C Vallières A Guay B Ivers H Savard J Mérette C cognitive behavioral therapy, singly and combined with medication, for persistent insomnia a randomized controlled trial. *JAMA.* (2009) 301:2005–15. 10.1001/jama.2009.682 19454639 PMC3050624

[64] Hao F Tan W Jiang L Zhang L Zhao X Zou Y Do psychiatric patients experience more psychiatric symptoms during COVID-19 pandemic and lockdown? A case-control study with service and research implications for immunopsychiatry. *Brain Behav Immun.* (2020) 87:100–6. 10.1016/j.bbi.2020.04.069 32353518 PMC7184991

[65] Trauer J Qian M Doyle J Rajaratnam S Cunnington D. Cognitive behavioral therapy for chronic insomnia: a systematic review and meta-analysis. *Ann Intern Med.* (2015) 163:191–204. 10.7326/M14-2841 26054060

[66] Morin C Rodrigue S Ivers H. Role of stress, arousal, and coping skills in primary insomnia. *Psychosom Med.* (2003) 65:259–67. 10.1097/01.PSY.0000030391.09558.A3 12651993

[67] Daley M Morin C LeBlanc M Grégoire J Savard J. The economic burden of insomnia: direct and indirect costs for individuals with insomnia syndrome, insomnia symptoms, and good sleepers. *Sleep.* (2009) 32:55–64. 19189779 PMC2625324

[68] Sateia M Buysse D Krystal A Neubauer D Heald J. Clinical practice guideline for the pharmacologic treatment of chronic insomnia in adults: an American academy of sleep medicine clinical practice guideline. *J Clin Sleep Med.* (2017) 13:307–49. 10.5664/jcsm.6470 27998379 PMC5263087

[69] National Institutes of Health. National institutes of health state of the science conference statement–manifestations and management of chronic insomnia in adults June 13-15, 2005. *Sleep.* (2005) 28:1049–57. 10.1093/sleep/28.9.1049 16268373

[70] Edinger J Wohlgemuth W Radtke R Marsh G Quillian R. Cognitive behavioral therapy for treatment of chronic primary insomnia–a randomized controlled trial. *JAMA.* (2001) 285:1856–64. 10.1001/jama.285.14.1856 11308399

[71] Riemann D Voderholzer U. Primary insomnia: a risk factor to develop depression? *J Affect Disord.* (2003) 76:255–9. 10.1016/S0165-0327(02)00072-1 12943956

[72] Neckelmann D Mykletun A Dahl A. Chronic insomnia as a risk factor for developing anxiety and depression. *Sleep.* (2007) 30:873–80. 10.1093/sleep/30.7.873 17682658 PMC1978360

[73] Fortier-Brochu E Beaulieu-Bonneau S Ivers H Morin C. Insomnia and daytime cognitive performance: a meta-analysis. *Sleep Med Rev.* (2012) 16:83–94. 10.1016/j.smrv.2011.03.008 21636297

[74] Vgontzas AN Fernandez-Mendoza J Liao D Bixler EO. Insomnia with objective short sleep duration: the most biologically severe phenotype of the disorder. *Sleep Med Rev.* (2013) 17:241–54. 10.1016/j.smrv.2012.09.005 23419741 PMC3672328

[75] Lichstein K Stone K Donaldson J Nau S Soeffing J Murray D Actigraphy validation with insomnia. *Sleep.* (2006) 29:232–9. 16494091

[76] Morin CM Vallières A Hans I. Dysfunctional beliefs and attitudes about sleep (DBAS): validation of a brief version (DBAS-16). *Sleep.* (2011) 30:1547–54. 10.1093/sleep/30.11.1547 18041487 PMC2082102

[77] Stepanski E Wyatt J. Use of sleep hygiene in the treatment of insomnia. *Sleep Med Rev.* (2003) 7:215–25. 10.1053/smrv.2001.0246 12927121

[78] Altena E Baglioni C Espie CA Ellis J Gavriloff D Holzinger B Dealing with sleep problems during home confinement due to the COVID-19 outbreak: practical recommendations from a task force of the European CBT-I Academy. *J Sleep Res.* (2020) 29:e13052. 10.1111/jsr.13052 32246787

[79] Morphy H Dunn K Lewis M Boardman H Croft P. Epidemiology of insomnia: a longitudinal study in a UK population. *Sleep.* (2007) 30:274–80. 17425223

[80] Sivertsen B Omvik S Pallesen S Bjorvatn B Havik O Kvale G Cognitive behavioral therapy vs zopiclone for treatment of chronic primary insomnia in older adults–a randomized controlled trial. *JAMA.* (2006) 295:2851–8. 10.1001/jama.295.24.2851 16804151

[81] Espie C. Insomnia: conceptual issues in the development, persistence, and treatment of sleep disorder in adults. *Annu Rev Psychol.* (2002) 53:215–43. 10.1146/annurev.psych.53.100901.135243 11752485

[82] Johnson E Roth T Schultz L Breslau N. Epidemiology of DSM-IV insomnia in adolescence: lifetime prevalence, chronicity, and an emergent gender difference. *Pediatrics.* (2006) 117:e247–56. 10.1542/peds.2004-2629 16452333

[83] Bertolazi A Fagondes S Hoff L Dartora E Miozzo I de Barba M Validation of the Brazilian Portuguese version of the Pittsburgh sleep quality index. *Sleep Med.* (2011) 12:70–5. 10.1016/j.sleep.2010.04.020 21145786

[84] Irwin M Cole J Nicassio P. Comparative meta-analysis of behavioral interventions for insomnia and their efficacy in middle-aged adults and in older adults 55+years of age. *Health Psychol.* (2006) 25:3–14. 10.1037/0278-6133.25.1.3 16448292

[85] Jacobs G Pace-Schott E Stickgold R Otto M. Cognitive behavior therapy and pharmacotherapy for insomnia– randomized controlled trial and direct comparison. *Arch Intern Med.* (2004) 164:1888–96. 10.1001/archinte.164.17.1888 15451764

[86] Soldatos C Dikeos D Paparrigopoulos T. The diagnostic validity of the athens insomnia scale. *J Psychosom Res.* (2003) 55:263–7. 10.1016/S0022-3999(02)00604-9 12932801

[87] Jansson-Frojmark M Lindblom K. A bidirectional relationship between anxiety and depression, and insomnia? A prospective study in the general population. *J Psychosom Res.* (2008) 64:443–9. 10.1016/j.jpsychores.2007.10.016 18374745

[88] Krystal A Walsh J Laska E Caron J Amato D Wessel T Sustained efficacy of eszopiclone over 6 months of nightly treatment: results of a randomized, double-blind, placebo-controlled study in adults with chronic insomnia. *Sleep.* (2003) 26:793–9. 10.1093/sleep/26.7.793 14655910

[89] Prober D Rihel J Onah A Sung R Schier A. Hypocretin/orexin overexpression induces an insomnia-like phenotype in zebrafish. *J Neurosci.* (2006) 26:13400–10. 10.1523/JNEUROSCI.4332-06.2006 17182791 PMC6675014

[90] Savard J Simard S Ivers H Morin C. Randomized study on the efficacy of cognitive-bahavioural therapy for insomnia secondary to breast cancer, part I: Sleep and psychological effects. *J Clin Oncol.* (2005) 23:6083–96. 10.1200/JCO.2005.09.548 16135475

[91] Youngstedt S. Effects of exercise on sleep. *Clin Sports Med.* (2005) 24:355–65,xi. 10.1016/j.csm.2004.12.003 15892929

[92] Fava M McCall W Krystal A Wessel T Rubens R Caron J Eszopiclone co-administered with fluoxetine in patients with insomnia coexisting with major depressive disorder. *Biol Psychiatry.* (2006) 59:1052–60. 10.1016/j.biopsych.2006.01.016 16581036

[93] Vgontzas A Liao D Pejovic S Calhoun S Karataraki M Bixler E. Insomnia with objective short sleep duration is associated with type 2 diabetes a population-based study. *Diabetes Care.* (2009) 32:1980–5. 10.2337/dc09-0284 19641160 PMC2768214

[94] Morin CM Bélanger L LeBlanc M Ivers H Savard J Espie C The natural history of insomnia: a population-based 3-year longitudinal study. *Arch Intern Med.* (2009) 169:447–53. 10.1001/archinternmed.2008.610 19273774

[95] Perlis M Smith M Andrews P Orff H Giles D. Beta/gamma EEG activity in patients with primary and secondary insomnia and good sleeper controls. *Sleep.* (2001) 24:110–7. 10.1093/sleep/24.1.110 11204046

[96] Leger D Guilleminault C Bader G Lévy E Paillard M. Medical and socio-professional impact of insomnia. *Sleep.* (2002) 25:625–9. 10.1093/sleep/25.6.621 12224841

[97] Sofi F Cesari F Casini A Macchi C Abbate R Gensini G. Insomnia and risk of cardiovascular disease: a meta-analysis. *Eur J Prev Cardiol.* (2014) 21:57–64. 10.1177/2047487312460020 22942213

[98] Buysse D Germain A Moul D Franzen P Brar L Fletcher M Efficacy of brief behavioral treatment for chronic insomnia in older adults. *Arch Intern Med.* (2011) 171:887–95. 10.1001/archinternmed.2010.535 21263078 PMC3101289

[99] LeBlanc M Mérette C Savard J Ivers H Baillargeon L Morin C. Incidence and risk factors of insomnia in a population-based sample. *Sleep.* (2009) 32:1027–37. 10.1093/sleep/32.8.1027 19725254 PMC2717193

[100] Wilson S Anderson K Baldwin D Dijk D Espie A Espie C British Association for Psychopharmacology consensus statement on evidence-based treatment of insomnia, parasomnias and circadian rhythm disorders. *J Psychopharmacol.* (2010) 33:923–47. 10.1177/0269881119855343 31271339

[101] Palesh O Roscoe J Mustian K Roth T Savard J Ancoli-Israel S Prevalence, demographics, and psychological associations of sleep disruption in patients with cancer: university of rochester cancer center-community clinical oncology program. *J Clin Oncol.* (2010) 28:292–8. 10.1200/JCO.2009.22.5011 19933917 PMC2815717

[102] Kessler R Berglund P Coulouvrat C Hajak G Roth T Shahly V Insomnia and the performance of US workers: results from the America insomnia survey. *Sleep.* (2011) 34:1161–71. 10.5665/SLEEP.1230 21886353 PMC3157657

[103] Reid K Baron K Lu B Naylor E Wolfe L Zee P. Aerobic exercise improves self-reported sleep and quality of life in older adults with insomnia. *Sleep Med.* (2010) 11:934–40. 10.1016/j.sleep.2010.04.014 20813580 PMC2992829

[104] Espie C Kyle S Williams C Ong J Douglas N Hames P A randomized, placebo-controlled trial of online cognitive behavioral therapy for chronic insomnia disorder delivered via an automated media-rich web application. *Sleep.* (2012) 35:769–81. 10.5665/sleep.1872 22654196 PMC3353040

[105] Harrison T Keating G. Zolpidem–a review of its use in the management of insomnia. *CNS Drugs.* (2005) 19:65–89. 10.2165/00023210-200519010-00008 15651908

[106] Zachariae R Lyby M Ritterband L O’Toole M. Efficacy of internet-delivered cognitive-behavioral therapy for insomnia–a systematic review and meta-analysis of randomized controlled trials. *Sleep Med Rev.* (2016) 30:1–10. 10.1016/j.smrv.2015.10.004 26615572

[107] Ford DE Kamerow DB. Epidemiologic study of sleep disturbances and psychiatric disorders. An opportunity for prevention? *JAMA*. (1989) 262:1479–84. 10.1001/jama.262.11.1479 2769898

[108] Breslau N Roth T Rosenthal L Andreski P. Sleep disturbance and psychiatric disorders: a longitudinal epidemiological study of young adults. *Biol Psychiatry*. (1996) 39:411–8. 10.1016/0006-3223(95)00188-3 8679786

[109] Guo J Gu D Zhao T Zhao Z Xiong Y Sun M Trends in piezo channel research over the past decade: a bibliometric analysis. *Front Pharmacol.* (2021) 12:668714. 10.3389/fphar.2021.668714 33935792 PMC8082452

[110] Ji X Ivers H Beaulieu-Bonneau S Morin C. Complementary and alternative treatments for insomnia/insomnia -depression-anxiety symptom cluster: meta-analysis of English and Chinese literature. *Sleep Med Rev.* (2021) 58:101445. 10.1016/j.smrv.2021.101445 33582583

[111] Selvanathan J Pham C Nagappa M Peng PW Englesakis M Espie CA Cognitive behavioral therapy for insomnia in patients with chronic pain–a systematic review and meta-analysis of randomized controlled trials. *Sleep Med Rev.* (2021) 60:101460. 10.1016/j.smrv.2021.101460 33610967

[112] Lancee J Harvey AG Morin CM Ivers H Zweerde T v Blanken TF. Network intervention analyses of cognitive therapy and behavior therapy for insomnia: symptom specific effects and process measures. *Behav Res Ther.* (2022) 153:104100. 10.1016/j.brat.2022.104100 35462241

[113] Ritterband LM Thorndike FP Morin CM Gerwien R Enman NM Xiong R Real-world evidence from users of a behavioral digital therapeutic for chronic insomnia. *Behav Res Ther.* (2022) 153:104084. 10.1016/j.brat.2022.104084 35405424

[114] Morin CM Bjorvatn B Chung F Holzinger B Partinen M Penzel T Insomnia, anxiety, and depression during the COVID-19 pandemic: an international collaborative study. *Sleep Med.* (2021) 87:38–45. 10.1016/j.sleep.2021.07.035 34508986 PMC8425785

[115] Manzar M Jahrami H Bahammam A. Structural validity of the insomnia severity index: a systematic review and meta-analysis. *Sleep Med Rev.* (2021) 60:101531. 10.1016/j.smrv.2021.101531 34428679

[116] Spielman A Caruso L Glovinsky P. A behavior perspective on insomnia treatment. *Psychiatr Clin North Am.* (1987) 10:541–53. 10.1016/S0193-953X(18)30532-X 3332317

[117] Su T Lien T. Prevalence of psychiatric morbidity and psychological adaptation of the nurses in a structured SARS caring unit during outbreak: a prospective and periodic assessment study in Taiwan. *J Psychiatr Res.* (2007) 41:119–30. 10.1016/j.jpsychires.2005.12.006 16460760 PMC7094424

[118] Li Y Chen B Hong Z Sun Q Dai Y Basta M Insomnia symptoms during the early and late stages of the COVID-19 pandemic in China: a systematic review and meta-analysis. *Sleep Med.* (2022) 91:262–72. 10.1016/j.sleep.2021.09.014 34732293 PMC8479411

[119] Šljivo A Juginovi A Ivanovi K Quraishi I Mula A Kovaèevi Z Sleep quality and patterns of young West Balkan adults during the third wave of COVID-19 pandemic: a cross-sectional study. *BMJ Open.* (2022) 12:e060381. 10.1136/bmjopen-2021-060381 35613815 PMC9174533

[120] Conte F De Rosa O Rescott ML Arabia TP D’Onofrio P Lustro A High sleep fragmentation parallels poor subjective sleep quality during the third wave of the Covid-19 pandemic: an actigraphic study. *J Sleep Res.* (2022) 31:e13519. 10.1111/jsr.13519 34797004 PMC8646572

[121] Taylor D Lichstein K Durrence H. Insomnia as a health risk factor. *Behav Sleep Med.* (2003) 1:227. 10.1207/S15402010BSM0104_5 15600216

[122] Sforza M Galbiati A Zucconi M Casoni F Hensley M Ferini-Strambi L Depressive and stress symptoms in insomnia patients predict group cognitive-behavioral therapy for insomnia long-term effectiveness: a data-driven analysis. *J Affect Disord.* (2021) 289:117–24. 10.1016/j.jad.2021.04.021 33979721

[123] Esposito E Di Matteo V Di Giovanni G. Serotonin-dopamine interaction: an overview. *Prog Brain Res.* (2008) 172:3–6. 10.1016/S0079-6123(08)00901-1 18772025

[124] Hamann C Bankmann J Mora Maza H Kornhuber J Zoicas I Schmitt-Böhrer A. Social fear affects limbic system neuronal activity and gene expression. *Int J Mol Sci.* (2022) 23:8228. 10.3390/ijms23158228 35897794 PMC9367789

[125] Blake M Trinder J Allen N. Mechanisms underlying the association between insomnia, anxiety, and depression in adolescence: implications for behavioral sleep interventions. *Clin Psychol Rev.* (2018) 63:25–40. 10.1016/j.cpr.2018.05.006 29879564

[126] Sabbah S Worden MS Laniado DD Berson DM Sanes JN. Luxotonic signals in human prefrontal cortex as a possible substrate for effects of light on mood and cognition. *Proc Natl Acad Sci USA.* (2022) 119:e2118192119. 10.1073/pnas.2118192119 35867740 PMC9282370

[127] Liverant G Arditte Hall K Wieman S Pineles S Pizzagalli D. Associations between insomnia and reward learning in clinical depression. *Psychol Med.* (2021) 26:1–10. 10.1017/S003329172100026X 33634765

[128] Wieman ST Hall KA MacDonald HZ Gallagher MW Suvak MK Rando AA Relationships among sleep disturbance, reward system functioning, anhedonia, and depressive symptoms. *Behav Ther.* (2022) 53:105–18. 10.1016/j.beth.2021.06.006 35027152

[129] Schiller CE Walsh E Eisenlohr-Moul TA Prim J Dichter GS Schiff L Effects of gonadal steroids on reward circuitry function and anhedonia in women with a history of postpartum depression. *J Affect Disord.* (2022) 314:176–84. 10.1016/j.jad.2022.06.078 35777494 PMC9605402

[130] Kirschner M Cathomas F Manoliu A Habermeyer B Simon JJ Seifritz E Shared and dissociable features of apathy and reward system dysfunction in bipolar I disorder and schizophrenia. *Psychol Med.* (2020) 50:936–47. 10.1017/S0033291719000801 30994080

[131] Bjorvatn B Jernelöv S Pallesen S. Insomnia–a heterogenic disorder often comorbid with psychological and somatic disorders and diseases: a narrative review with focus on diagnostic and treatment challenges. *Front Psychol.* (2021) 12:639198. 10.3389/fpsyg.2021.639198 33643170 PMC7904898

[132] Fietze I Laharnar N Koellner V Penzel T. The different faces of insomnia. *Front Psychiatry.* (2021) 12:683943. 10.3389/fpsyt.2021.683943 34267688 PMC8276022

[133] Reynolds CF III Kupfer DJ Buysse DJ Coble PA Yeager A. Subtyping DSM-III-R primary insomnia: a literature review by the DSM-IV work group on sleep disorders. *Am J Psychiatry.* (1991) 148:432–8. 10.1176/ajp.148.4.432 2006686

[134] American Academy of Sleep Medicine. *International Classification of Sleep Disorders.* 3rd ed. Darien, IL: American Academy of Sleep Medicine (2014).

[135] American Academy of Sleep Medicine. *International Classification of Sleep Disorders.* 2nd ed. Darien, IL: American Academy of Sleep Medicine (2005).

[136] Blanken T Benjamins J Borsboom D Vermunt J Paquola C Ramautar J Insomnia disorder subtypes derived from life history and traits of affect and personality. *Lancet Psychiatry.* (2019) 6:151–63. 10.1016/S2215-0366(18)30464-4 30630691

[137] Benjamins JS Migliorati F Dekker K Wassing R Moens S Blanken TF Insomnia heterogeneity: characteristics to consider for data-driven multivariate subtyping. *Sleep Med. Rev.* (2017) 36:71–81. 10.1016/j.smrv.2016.10.005 29066053

[138] Dang-Vu TT Hatch B Salimi A Mograss M Boucetta S O’Byrne J Sleep spindles may predict response to cognitive-behavioral therapy for chronic insomnia. *Sleep Med.* (2017) 39:54–61. 10.1016/j.sleep.2017.08.012 29157588

[139] van de Laar M Leufkens T Bakker B Pevernagie D Overeem S. Phenotypes of sleeplessness: stressing the need for psychodiagnostics in the assessment of insomnia. *Psychol Heal Med.* (2017) 22:902–10. 10.1080/13548506.2017.1286360 28133972

[140] Baglioni C Regen W Teghen A Spiegelhalder K Feige B Nissen C Sleep changes in the disorder of insomnia: a meta-analysis of polysomnographic studies. *Sleep Med Rev.* (2014) 18:195–213. 10.1016/j.smrv.2013.04.001 23809904

[141] Van Someren EJW Altena E Ramauter JR Stoffers D Benjamins J Moens S *Imaging Causes and Consequences of Insomnia and Sleep Complaints.* Cambridge: Cambridge University Press (2013). p. 187–96.

[142] Harvey C Gehrman P Espie C. Who is predisposed to insomnia: a review of familial aggregation, stress-reactivity, personality and coping style. *Sleep Med Rev.* (2014) 18:237–47. 10.1016/j.smrv.2013.11.004 24480386

[143] Okajima I Komada Y Inoue Y. A meta-analysis on the treatment effectiveness of cognitive behavioral therapy for primary insomnia. *Sleep Biol Rhythms.* (2011) 9:24–34. 10.1111/j.1479-8425.2010.00481.x

[144] Randall C Nowakowski S Ellis JG. Managing acute insomnia in prison: evaluation of a “One-Shot” cognitive behavioral therapy for insomnia (CBT-I) intervention. *Behav Sleep Med.* (2019) 17:827–36. 10.1080/15402002.2018.1518227 30289290

[145] Luik A van der Zweerde T van Straten A Lancee J. Digital delivery of cognitive behavioral therapy for insomnia. *Curr Psychiatry Rep.* (2019) 21:50. 10.1007/s11920-019-1041-0 31161406 PMC6546653

[146] Vedaa Ø Kallestad H Scott J Smith O Pallesen S Morken G Effects of digital cognitive behavioral therapy for insomnia on insomnia severity: a large-scale randomised controlled trial. *Lancet Digit Health.* (2020) 2:e397–406. 10.1016/S2589-7500(20)30135-7 33328044

[147] Liu X Li Y Yan R Timo H Li D Liu S The platform development, adherence and efficacy to a digital Brief therapy for insomnia (dBTI) during the COVID-19 pandemic. *Methods.* (2022) 205:39–45. 10.1016/j.ymeth.2022.04.016 35526723 PMC9070004

[148] Léger D Debellemaniere E Rabat A Bayon V Benchenane K Chennaoui M. Slow-wave sleep: from the cell to the clinic. *Sleep Med Rev.* (2018) 41:113–32. 10.1016/j.smrv.2018.01.008 29490885

[149] Rasch B Born J. About sleeps role in memory. *Physiol Rev.* (2013) 93:681–766. 10.1152/physrev.00032.2012 23589831 PMC3768102

[150] Ong J Patanaik A Chee N Lee X Poh J Chee M. Auditory stimulation of sleep slow oscillations modulates subsequent memory encoding through altered hippocampal function. *Sleep (Basel).* (2018) 5:1–11. 10.1093/sleep/zsy031 29425369 PMC5946855

[151] Marshall L. Transcranial direct current stimulation during sleep improves declarative memory. *J Neurosci.* (2004) 44:9985–92. 10.1523/JNEUROSCI.2725-04.2004 15525784 PMC6730231

[152] Walsh J. Enhancement of slow wave sleep: implications for insomnia. *J Clin Sleep Med.* (2009) 5(Suppl. 2):S27–32. 10.5664/jcsm.5.2S.S27 19998872 PMC2824211

[153] Zhang Y Gruber R. Can slow-wave sleep enhancement improve memory? A review of current approaches and cognitive outcomes. *Yale J Biol Med.* (2019) 92:63–80. 30923474 PMC6430170

[154] Scholes S Santisteban J Zhang Y Bertone A Gruber R. Modulation of Slow-wave sleep: implications for psychiatry. *Curr Psychiatry Rep.* (2020) 22:52. 10.1007/s11920-020-01175-y 32710222

[155] Wood KH Memon AA Memon RA Joop A Pilkington J Catiul C Slow wave sleep and EEG delta spectral power are associated with cognitive function in Parkinson’s disease. *J Parkinsons Dis.* (2021) 11:703–14. 10.3233/JPD-202215 33361608 PMC8058231

[156] Facchin L Schöne C Mensen A Bandarabadi M Pilotto F Saxena S Slow waves promote sleep-dependent plasticity and functional recovery after stroke. *J Neurosci.* (2020) 40:8637–51. 10.1523/JNEUROSCI.0373-20.2020 33087472 PMC7643301

[157] Besedovsky L Cordi M Wißlicen L Martínez-Albert E Born J Rasch B. Hypnotic enhancement of slow-wave sleep increases sleep-associated hormone secretion and reduces sympathetic predominance in healthy humans. *Commun Biol.* (2022) 5:747. 10.1038/s42003-022-03643-y 35882899 PMC9325885

